# Increased Cytotoxicity of Vanadium to CHO-K1 Cells in the Presence of Inorganic Selenium

**DOI:** 10.1007/s00128-015-1615-4

**Published:** 2015-07-23

**Authors:** Iwona Zwolak

**Affiliations:** Department of Cell Biology, Institute of Environmental Protection, The John Paul II Catholic University of Lublin, Kraśnicka Ave. 102, 20-718 Lublin, Poland

**Keywords:** Selenium, Vanadium, Interaction, Cytotoxicity, CHO-K1 cells

## Abstract

The effect of selenium applied as sodium selenite (Na_2_SeO_3_) on the cytotoxicity of vanadyl sulphate (VOSO_4_) was examined using CHO-K1 cells. From the resazurin-based assay, it appears that Na_2_SeO_3_ at low doses (0.5 and 1 μM) can enhance 100 μM VOSO_4_-induced cell damage. The two-way ANOVA analysis revealed that the increased cell damage was a consequence of a synergistic interaction of 0.5 μM Na_2_SeO_3_ with VOSO_4_ and 1 μM Na_2_SeO_3_ with VOSO_4_. Observations performed with a phase-contrast microscope showed most cells to be rounded upon treatment with VOSO_4_ alone. In turn, a majority of cells co-treated with VOSO_4_ and 1 μM Na_2_SeO_3_ were elongated, and exhibited cytoplasmic vacuolization. These results warn of the potential contribution of inorganic selenium to vanadium-induced toxicity.

Vanadium (V) is a trace element in human tissues. So far, no essential functions have been identified for V in higher animals and people. However, due to the wide and growing use of this element in industry, V is regarded as an occupational/environmental pollutant (Fortoul et al. 2014). People can be exposed to V as a result of combustion of fossil fuels (vanadium is a natural component of coal, oil), VO_5_ manufacturing, processing of V-rich steel alloy, cleaning/repair of oil-fired boilers, or handling of catalysts for chemical production (Fortoul et al. 2014; Zwolak 2014a). Vanadium poisoning of cattle via ingestion of V-polluted grass has been documented in South Africa (McCrindle et al. 2001; Gummow et al. 2006). Also, there are reports describing adverse V effects on human health associated with occupational/environmental exposure via inhalation (Ehrlich et al. 2008; Patel et al. 2009; Zwolak 2014a).

Another element studied here, selenium (Se), is a well-known micronutrient whose both beneficial and toxic attributes raise a lot of interest among researchers (reviewed by Letavayová et al. 2006; Schrauzer 2009; Rayman 2012; Vinceti et al. 2013a, b). Similar to V, Se can also pose a risk as an environmental pollutant. As reviewed by Lemly (2004), some human activities like coal mining and combustion, metal mining, oil industry or agricultural irrigation can contribute to excessive release of Se to the aquatic environment. Waterborne Se easily bioaccumulates in the food chain and thus even a slight increase in the water concentration of this element threatens fish and wildlife (Lemly 2004). Excessive intake of Se from dietary sources was shown to induce deleterious health effects in animals and people (Zwolak and Zaporowska 2012). On the other hand, there are research studies available that have shown that both V and Se can exert many biological actions with potential pharmacological applications such as insulin-mimetic and osteogenic activity (V), or antitumor effects (V and Se) (Cortizo et al. 2006; Molinuevo et al. 2008; Rayman 2012; Rehder 2012). Notably, both the form of Se, i.e. sodium selenite, and the form of V, vanadyl sulphate, used for the current study are available to the public as dietary supplements either singly or in combination (Clarkson and Rawson 1999; Rayman 2012). In view of these observations, the research experiments presented in this paper on potential interactive effects between Se and V may be of value.

As recently published, the toxicity of inorganic V in mammals can be affected in the presence of certain essential elements. For example, magnesium has been found to depress the adverse outcomes of V in rats (Ścibior et al. 2013, 2014). In turn, another essential element, iron, exacerbated the V-induced cytotoxicity in vitro (Todorich et al. 2011). The interaction between V and Se, which has been studied here, has been poorly examined. An early in vivo study by Haider et al. (1998) reported that prooxidant effects of V in rats can be reduced by Se. However, previous work from our laboratory demonstrated that adverse actions caused by sodium metavanadate in mouse 3T3/BALB fibroblasts could not be prevented by 24 h pre- or coexposure with sodium selenite (Zwolak and Zaporowska 2009, 2010). Taking into account the dissimilar results between Haider’s and our studies, we decided to continue the examination of the V–Se interaction. In the present work, we coexposed the CHO-K1 cells to vanadyl sulphate (VOSO_4_) and sodium selenite (Na_2_SeO_3_) for 48 h (instead of 24 h in the previous work) to show that the time of coexposure of CHO-K1 cells to vanadium and selenium in the experimental conditions applied in this study may have importance in showing interactive effects of these elements on cell damage.

The biological interaction resulting from a simultaneous exposure of the organism to two (or more) factors is the type of effect that could not be expected from the addition of individual responses to each factor, in contrast to the additive effect, which is the sum of responses to each factor. There can be two types of biological interactions: synergistic, which results in amplification of the additive effect, and antagonistic, when the additive effect is reduced (Dunne 2010). The two-way ANOVA represents a statistical tool that allows detection of the interaction between factors examined (in this paper these are vanadyl and selenite). Notably, this statistical analysis has already enabled researchers to show interactive effects of V with chromium (Ścibior et al. 2010) and V with magnesium (Ścibior et al. 2013) on oxidative stress markers in rats. By using the same statistics, the present study reveals that Se given as selenite can synergistically potentiate vanadyl-induced cell damage in a CHO-K1 cell culture model.

## Materials and Methods

Dulbecco’s Modified Eagle’s Medium (DMEM), foetal bovine serum (FBS), and the antibiotic–antimycotic mixture were purchased from PAA Laboratories Gmbh (Pasching, AT). Resazurin test, sodium selenite (Na_2_SeO_3_), and vanadyl sulphate hydrate (VOSO_4_·xH_2_O) were purchased from Sigma–Aldrich (St Louis, MO, USA). Trypsin solution (0.25 %) was ordered at Biomed, Lublin, PL.

Chinese hamster ovary cells (CHO-K1 line) were a kind gift from the Department of Cell Biology and Electron Microscopy (Institute of Biology, Jan Kochanowski University of Humanities and Sciences in Kielce, PL). The cells were cultivated in DMEM containing 5 % FBS, 100 U/mL penicillin, 100 μg/mL streptomycin, and 0.25 μg/mL amphotericin B in an incubator at 37°C and 5 % CO_2_. The cultures were passaged twice a week.

For the selenite and vanadyl treatment, the CHO-K1 cells (1 × 10^5^ cells/mL) were seeded on 96-well microplates in DMEM-5 % FBS and maintained at 37°C in 5 % CO_2_. The cells were allowed to attach for 2 h. Then, the medium was removed and DMEM-1 % FBS was added. 24 h after plating, the medium was replaced with fresh DMEM (with 1 % FBS) containing vanadyl sulphate (100 μM) and selenite (0.1, 0.5 or 1 μM) and the culture was continued for 48 h. The concentrations of vanadyl and selenite were chosen on the basis of our earlier investigations (Zwolak and Zaporowska 2009; Zwolak 2014b). After vanadyl and selenite exposure, the cytotoxicity was assessed using the resazurin test as described below.

The principle of the resazurin assay is based on the reduction of the blue resazurin dye to red resorufin in the presence of mitochondrial enzymes of metabolically active cells (Vega-Avila and Pungsley 2011). The blue dye resazurin absorbs light at a wavelength of 600 nm. The amount of resazurin in the culture medium that has not been converted to resorufin is proportional to the number of injured cells, which is accompanied by an increase in absorbance at 600 nm when compared to the control. Our earlier studies described this assay as a convenient and reliable method to measure viability of CHO-K1 cells after vanadate or vanadyl exposure (Zwolak 2013, 2014b).

For the assay, the treatment medium, which contained vanadyl or/and selenite, was changed to fresh DMEM (without FBS) and 10 μl of resazurin was added to each well. The plates were incubated at 37°C in humidified atmosphere of 5 % CO_2_ for 3 h. Then, the absorbance was read at 600 nm using a microplate spectrophotometer. Five independent experiments were performed with three wells per each treatment condition.

The data from the resazurin assay are presented as a percentage of untreated control cells and they were calculated as follows: A_test_/A_control_ × 100 % (A_test_: absorbance of cells treated with vanadyl or/and selenite, A_control_: absorbance of control cells). In the resazurin assay (colorimetric reading), cytotoxicity (or cell damage) is indicated by an increase in percentage values when compared to the control cells (cells which were not treated with vanadium or/and selenium). For statistical analysis, the absorbance values from five independent experiments were imported to the SPSS program. After the assurance of the normality of distribution, the two-way ANOVA was carried out to assess the influence of vanadyl, selenite, and their interaction (vanadyl × selenite) on the cytotoxicity. When a significant interactive effect between vanadyl and selenite was detected, subsequent calculations were performed in order to indicate the character of the interaction revealed, as described by Ścibior et al. (2012). Accordingly, when the simultaneous treatment of cells with vanadyl and selenite induced a cytotoxic effect that was greater (V + Se effect > V effect + Se effect) or smaller (V + Se effect < V effect + Se effect) than the sum of effects induced separately by vanadyl and selenite, the interaction was regarded as synergistic or antagonistic, respectively. One-way ANOVA followed by Dunnett’s T3 as a post hoc test was used in order to assess the differences between the means of the treatment groups. *p* < 0.05 was considered as significant.

## Results and Discussion

**Fig. 1:**
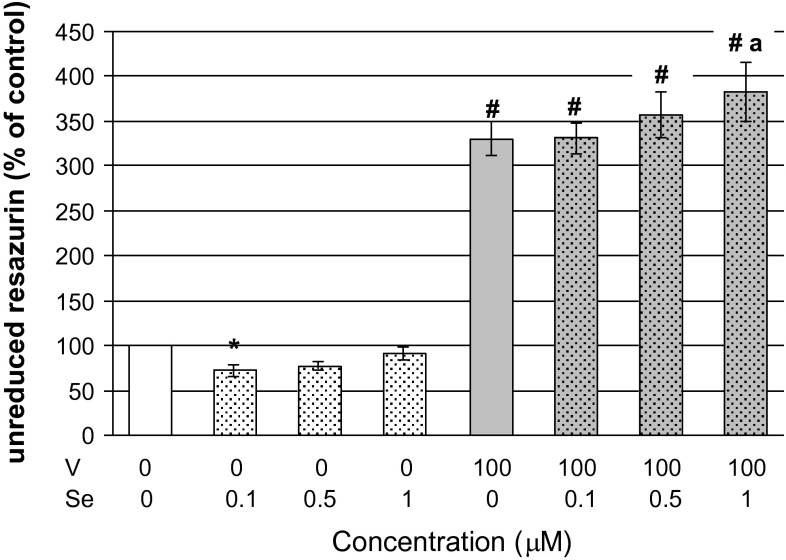
Cytotoxicity of V (VOSO_4_) and its combination with Se (Na_2_SeO_3_) to CHO-K1 cells as measured with the resazurin assay. The CHO-K1 cells were exposed to 100 μM VOSO_4_ in combination with Na_2_SeO_3_ (0.1, 0.5 or 1 μM) for 48 h and thereafter the cytotoxic effect was assessed with the resazurin test. In this test, the amount of resazurin that was not reduced to resorufin is proportional to the number of injured cells. The absorbance of resazurin in control cells (treated with neither V nor Se) was taken as 100 %. Results are presented as a percentage of control cells and represent mean ± SEM derived from five independent experiments. Differences between means (ANOVA/Dunnett’s T3 test) are indicated by *p < 0.05 significantly *lower* than control, ^#^ *p* < 0.001 significantly *higher* than control, ^a^ *p* < 0.05 significantly *higher* than V alone

The results of the resazurin assay obtained demonstrated that the 48-h exposure of CHO-K1 cells to 100 μM vanadyl alone induced significant (*p* < 0.05, one-way ANOVA with Dunnett’s T3 test) cytotoxicity compared to the control (cells treated with neither vanadyl nor selenite, Fig. 1). Treatment of cells only with selenite at concentrations of 0.1 and 0.5 μM seemed to slightly decrease cell damage compared to the control cultures, and the viability of cells exposed to 1 μM selenite was similar to the control. The three above-mentioned concentrations of selenite were regarded as non-cytotoxic. Importantly, the review of literature data revealed that cultured mammalian cells substantially differed in their sensitivity to selenite, as described hereafter. For instance, cultured human neuronal cells showed a viability decrease following exposure to selenite at doses as low as 0.1 μM (Maraldi et al. 2011). However, concentrations of selenite comparable to those used in our study were non-toxic to primary rabbit hepatocytes (Müller and Pallauf 2003), human hepatoma (HepG2) cells (Helmy et al. 2000), prostatic (PNT-1) cells (Maraldi et al. 2011), or human HaCaT keratinocytes (Hazane-Puch et al. 2013). Notably, human exposure to inorganic selenium via drinking water at concentrations similar to or much higher than those studied here have been documented in some regions of the world (reviewed by Vinceti et al. 2013a).

As presented in Fig. 1, vanadyl in the combination with 0.5 or 1 μM selenite induced stronger cytotoxic responses (by 26 % and 52 % respectively) than vanadyl alone, with the latter increase being statistically significant (*p* < 0.05, one-way ANOVA with Dunnett’s T3 test). The two-way ANOVA analysis (Table 1) revealed that the stronger cytotoxicity in cells co-treated with selenite and vanadyl was due to a synergistic interaction between vanadyl and 0.5 μM selenite (*p* = 0.034) and between vanadyl and 1 μM selenite (*p* = 0.002). Additionally, in the case of the combination of vanadyl with 1 μM selenite, independent action of 1 μM selenite (*p* = 0.006) also contributed to the increase in the vanadyl-induced cytotoxicity in the presence of selenite. On the other hand, as can be seen in Fig. 1, selenite at 0.1 μM did not affect vanadyl-induced toxicity. This shows a very fine threshold between the dose of selenite that does not interfere with vanadyl adverse effects (0.1 μM selenite) and the dose that can potentiate them (0.5 μM selenite). This certainly is in line with the notion that Se is an element with a very narrow safe range of exposure (Vinceti et al. 2013b). It is worth reminding that the detected interactive effects between vanadyl and selenite pertain to mitochondrial functions, since the in vitro assay with resazurin applied here records toxic effects of chemicals on the basis of mitochondrial enzyme activity.

**Table 1:** Results of two-way ANOVA on the effects of Na_2_SeO_3_ (Se), VOSO_4_ (V), and the interactive effects between Na_2_SeO_3_ and VOSO_4_ (V × Se) on CHO-K1 cell damage measured with a resazurin assay

Two-way anova analysis		Character of interaction
Main effect of V	F = 448.948, *p* = 0.000	–
Main effect of Se (0.1 μM)	NS	–
Interactive effect of V × Se (0.1 μM)	NS	–
Main effect of V	F = 728.505, *p* = 0.000	–
Main effect of Se (0.5 μM)	NS	–
Interactive effect of V × Se (0.5 μM)	F = 4.777, *p* = 0.034	Synergistic^a^
Main effect of V	F = 1110.036, *p* = 0.000	–
Main effect of Se (1.0 μM)	F = 8.249, *p* = 0.006	–
Interactive effect of V × Se (1.0 μM)	F = 10.578, *p* = 0.002	Synergistic^a^

*NS* no significant effect

^a^The effect of V in the presence of Se (V + Se effect) > sum of the effects of V and Se alone (Se effect + V effect) (synergistic interaction)

**Fig. 2:**
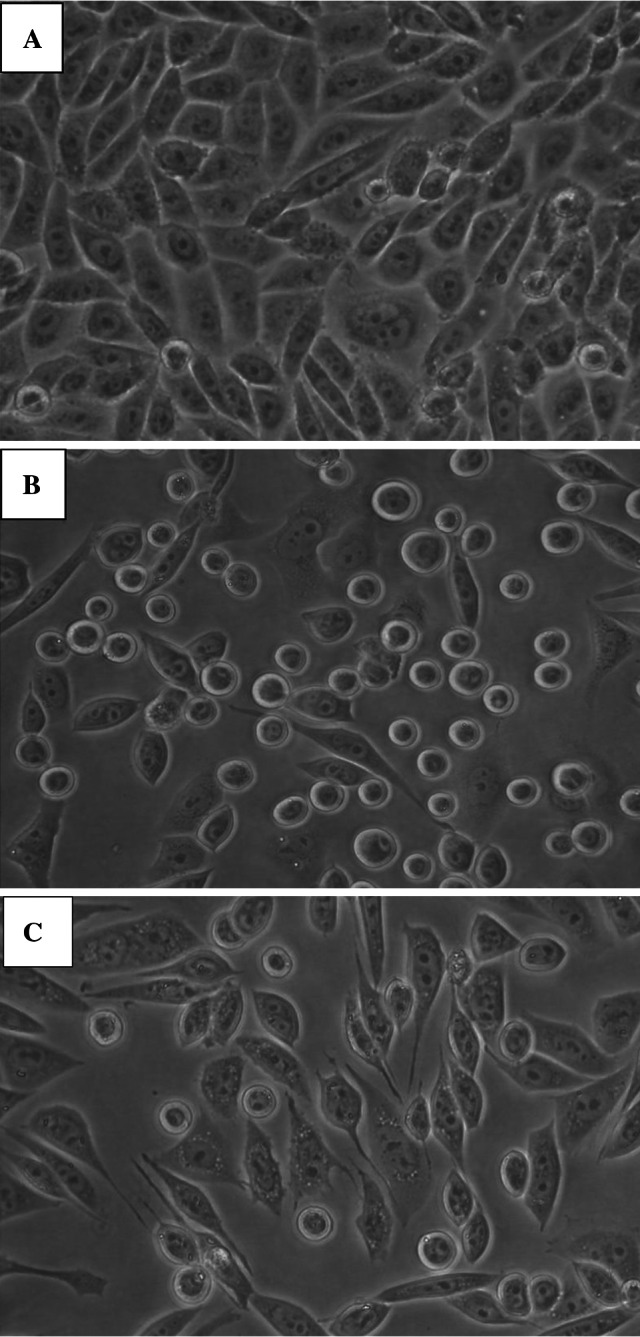
Morphology of CHO-K1 cells under the phase-contrast microscope. **a** Control cell culture, **b** cells after 48-h exposure to 100 μM VOSO_4_, **c** cells after 48-h exposure to the combination of 100 μM VOSO_4_ with 1 μM Na_2_SeO_3_

The analysis of cell morphology carried out under the phase-contrast microscope showed that the control CHO-K1 cell culture (at 72 h from seeding) developed a dense monolayer of cells with an epithelial-like morphology (Fig. 2a). The 48 h treatment with either of the selenite concentrations tested did not induce any changes in the appearance of the cells, compared to the untreated control cells (data not shown). Conversely, as expected, 100 μM vanadyl promoted significant changes in the appearance of the cells, compared to the control. In this treatment group, most cells were rounded and some cells were shrunk or spindle shaped (Fig. 2b). Additionally, nuclear chromatin condensation and marginalization was observed in the vanadyl-treated cells. As shown in Fig. 2c, cells exposed simultaneously to vanadyl and 1 μM selenite for 48 h obviously differed in their morphology from the vanadyl-only treated cells. Most cells were spread and elongated in shape and exhibited cytoplasmic vacuolization (Fig. 2c). The above-described changes were also observed in cultures treated with vanadyl and 0.5 μM selenite, but they were less intense. The morphology of the cells incubated with vanadyl and 0.1 μM selenite was comparable to that of the vanadyl-only treated cells (data not shown).

The phenomenon of cytoplasmic vacuolization of cultured cells following exposure to toxic compounds is generally seen as deterioration of cell functioning. These vacuoles may develop from different organelles such as Golgi elements, lysosomes, or mitochondria. Eventually, if the cytoplasmic vacuolization proceeds, it leads to destruction of cellular structures and cell death (Henics and Wheatley 1999). Summing up, the vacuolization of cells which were concomitantly treated with vanadyl and 1 μM selenite probably indicates an increase in cell damage in comparison with cells treated only with vanadyl, which would be in good agreement with the results obtained in the cytotoxicity assay. Still however, further microscopic analysis based on other methods is required to examine in more detail the apparent morphological differences between cells treated only with vanadyl and cells co-treated with vanadyl and 1 μM selenite.

The literature data on interaction between Se and V are sparse. The only study available demonstrated that Se in the form of SeO_2_ exhibited protective effects against metavanadate-induced neurotoxicity in rats (Haider et al. 1998), which is in sharp contrast with the current report. Probably, the design of experiments involving the use of specific research models or the kind of cells exposed may be attributed to the discrepancy between the results. Still however, the inconstancies between Haider’s et al. (1998) and our data show that the potential beneficial properties of inorganic Se during V-induced intoxication should be treated with caution. Some support for the current results comes from our earlier in vitro investigations in which metavanadate-induced cytotoxicity in mouse BALB/3T3 fibroblasts was not changed after 24-h co-treatment with 0.5 μM selenite (Zwolak and Zaporowska 2009). Similarly, under current experimental conditions, the 24-h co-exposure to vanadyl and 0.1–1 μM selenite did not modify the V-induced cell damage as shown by the resazurin assay and phase-contrast observations (data not shown). This however was not the case after the 48 h co-treatment, where a significant synergistic effect of the two tested doses of selenite (0.5 and 1 μM) on V-induced cell damage could be noticed. Hence, the time of co-exposure of cells to V and Se plays a critical role in demonstrating the interactive effects between the two elements.

So far, a number of human and animal/cell culture studies have provided evidence that Se (inorganic and organic forms) can be an effective countermeasure against the toxicity induced by other elements such as arsenic (As) or cadmium (Cd) (reviewed by Zwolak and Zaporowska 2012). The suggested mechanism of As and Cd detoxification is Se-dependent sequestration of these elements. Moreover, Se-dependent antioxidant enzymes such as glutathione peroxidase or thioredoxin reductase were also reported to play a significant role in the beneficial actions of Se against As or Cd. However, selenium potential to raise the toxicity of elements has also been documented in the literature. For instance, selenite increased the cytotoxicity of arsenite in primary rat hepatocytes (Styblo and Thomas 2001). In the report mentioned, the suggested mechanism of Se action was the inhibition of the detoxification pathway leading to increased retention of toxic arsenic forms in the cells examined. Recently, another Se compound, diphenyl diselenide, potentiated methylmercury neurotoxicity in rats (Dalla Corte et al. 2013). To the best of our knowledge, this study is the first to demonstrate that selenite can synergistically enhance the cytotoxicity of vanadyl. The mechanism of this interaction is unknown. Nevertheless, we cannot exclude that it may involve Se-dependent disturbance of cellular detoxification of vanadium excess. Synergistic toxicity between selenite and vanadyl may also be attributed to increased oxidative stress, as both compounds tested are known for their prooxidant activities (Bay et al. 1997, Yang et al. 2004, Chung et al. 2006).

In summary, by applying advanced statistical methods, this study is the first to show potentiation of vanadyl-induced cytotoxicity in the presence of selenite due to the synergistic interaction between vanadyl and selenite. Further, the increased cytotoxicity was accompanied by the appearance of specific morphological features such as cytoplasmic vacuolization suggesting deterioration of cell functioning. The preliminary data obtained here indicate that inorganic Se can interfere with the cytotoxic effects of V enhancing them through yet unknown mechanisms.
